# Brain Metabolism during Hallucination-Like Auditory Stimulation in Schizophrenia

**DOI:** 10.1371/journal.pone.0084987

**Published:** 2014-01-08

**Authors:** Guillermo Horga, Emilio Fernández-Egea, Anna Mané, Mireia Font, Kelly C. Schatz, Carles Falcon, Francisco Lomeña, Miguel Bernardo, Eduard Parellada

**Affiliations:** 1 Department of Psychiatry, New York State Psychiatric Institute, Columbia University Medical Center, New York, New York, United States of America; 2 Schizophrenia Unit, Neuroscience Institute, Hospital Clinic of Barcelona, Barcelona, Spain; 3 Department of Psychiatry and Clinical Psychobiology, University of Barcelona, Barcelona, Spain; 4 Department of Psychiatry, University of Cambridge, Cambridge, and the Cambridgeshire and Peterborough NHS Foundation Trust, Huntingdon, United Kingdom; 5 Centro de Investigación Biomédica en Red de Salud Mental (CIBERSAM), Madrid, Spain; 6 Centre Forum, Barcelona, Spain; 7 GIB-UB. CIBER-BBN, Barcelona, Spain; 8 Institut d′Investigacions Biomèdiques August Pi i Sunyer (IDIBAPS), Barcelona, Spain; University Of Cambridge, United Kingdom

## Abstract

Auditory verbal hallucinations (AVH) in schizophrenia are typically characterized by rich emotional content. Despite the prominent role of emotion in regulating normal perception, the neural interface between emotion-processing regions such as the amygdala and auditory regions involved in perception remains relatively unexplored in AVH. Here, we studied brain metabolism using FDG-PET in 9 remitted patients with schizophrenia that previously reported severe AVH during an acute psychotic episode and 8 matched healthy controls. Participants were scanned twice: (1) at rest and (2) during the perception of aversive auditory stimuli mimicking the content of AVH. Compared to controls, remitted patients showed an exaggerated response to the AVH-like stimuli in limbic and paralimbic regions, including the left amygdala. Furthermore, patients displayed abnormally strong connections between the amygdala and auditory regions of the cortex and thalamus, along with abnormally weak connections between the amygdala and medial prefrontal cortex. These results suggest that abnormal modulation of the auditory cortex by limbic-thalamic structures might be involved in the pathophysiology of AVH and may potentially account for the emotional features that characterize hallucinatory percepts in schizophrenia.

## Introduction

The pathophysiology of positive symptoms in schizophrenia remains a critical and poorly understood area of psychiatry. Auditory verbal hallucinations (AVH), a core positive symptom of schizophrenia, often manifest in the form of emotionally distressing voices. While several studies have successfully corroborated AVH-related activity in brain regions responsible for normal speech perception [1], little is known about the neural interface between emotion and perception as a potential mechanism for AVH, despite the rich emotional phenomenology that characterizes AVH.

Converging evidence indicates that the mesolimbic dopaminergic system, normally involved in reward prediction and assignment of salience to environmental stimuli, may be critically altered in individuals with schizophrenia [2]. Indeed, positive symptoms have been conceptualized as an usurpation of the normal process of salience attribution caused by dysregulation of mesolimbic dopamine, potentially leading to misattributed salience to internal representations and external stimuli [2].

Within the mesolimbic system, the amygdala plays a central role in recognizing emotionally meaningful events and their associations with sensory inputs, including fear conditioning [3]. Some amygdala nuclei interact with an array of cortical and subcortical structures including the striatum, midbrain dopaminergic system, and the prefrontal cortex (particularly its medial and orbital parts) to modulate reward processing by dynamically updating stimulus-value associations [4]. This physiological update of associations permits accurate predictions about the environment, and its malfunction has been proposed to lead to delusions and hallucinations [5]. Neuroimaging and molecular studies in schizophrenia have shown anomalies in constituents of the mesolimbic system. Meta-analyses have confirmed volume reductions in the amygdala and other limbic structures, some of which are already present at the onset of the disorder [6], [7]. Postmortem studies, in turn, indicate disturbances in amygdalocortical circuitry that are suggestive of increased excitatory afferents in the amygdalar projections to the neocortex [8].

Schizophrenia research has long used emotionally salient stimuli to probe for limbic dysfunction in schizophrenia during emotional challenge. Functional imaging studies using diverse visual paradigms of emotional challenge have revealed abnormal evoked responses in the amygdala of patients [9], [10], [11] as well as deficits in the normal influence of emotional salience on the visual cortex [12]. The influence of emotion on perception, specifically enhanced perception of aversive verbal material dependent on an intact left amygdala [13], is an intriguing mechanism with potential links to AVH generation. Auditory-emotional paradigms mimicking the aversive content of AVH are particularly appealing to evaluate the interface between perceptive and emotional facets of positive symptoms in schizophrenia. Studies using this type of auditory paradigm have reported increased hemodynamic response to emotional words in limbic and paralimbic regions, namely the amygdala, orbitofrontal cortex and cingulate gyrus, in chronic and remitted hallucinators [14], [15]. In addition to these abnormalities in evoked responses to emotional stimuli, patients with schizophrenia exhibit abnormalities in sustained activation and response habituation in mesolimbic structures such as the amygdala, hippocampus, and ventral striatum [16], [17], [18], although the relationship of abnormalities in this sustained, tonic activation and AVH is unclear. Tonic hyperactivity in mesolimbic structures could represent the stimulus-independent engagement of limbic circuitry proposed to be a crucial factor in the development of positive symptoms [2] and might account for modulatory failures in response to emotional cues [17]. To the best of our knowledge, the present study is the first to use fluorodeoxyglucose positron emission tomography (FDG-PET), which provides an index of sustained neural activity, during an emotional challenge in hallucination-prone patients with schizophrenia.

We aimed to investigate emotional and linguistic processing during hallucination-like auditory stimulation in remitted schizophrenia patients with AVH compared to healthy controls. FDG-PET measured accumulated neural activity at baseline and during the perception of aversive verbal stimuli mimicking AVH. We hypothesized that patients with schizophrenia would show an exaggerated sustained response to aversive verbal stimuli in limbic and auditory sensory regions (*hypothesis one*) as well as abnormal interactions between the amygdala and auditory cortex representing alterations in the neural interface between emotion and perception systems (*hypothesis two*).

## Methods

### Subjects

Nine patients meeting DSM-IV criteria for schizophrenia and eight matched healthy controls were recruited at the Hospital Clinic of Barcelona. All participants were right-handed. Only patients who reported prominent, commenting AVH during their first psychotic episode were prospectively included in this study. Additionally, psychotic symptoms remitted (PANSS-Positive score reduction >50% and complete remission of AVH, based on the PANSS item *hallucinatory behaviour*) after 4 weeks of treatment with risperidone 4–9 mg/day in all patients. Prior to this treatment, patients were antipsychotic-naïve. The current report focuses on the remission phase of AVH; FDG-PET findings during the acute psychotic episode have been previously reported for the same patients included here [19]. Diagnoses were established using the Structured Clinical Interview for DSM (SCIDDSM)-IV version and structured assessment with the Positive and Negative Syndrome Scale (PANSS) [20], and Comprehensive Assessment of Symptoms and History (CASH) [21]. Frequency and formal features of AVH during the acute psychotic episode were rated using the Psychotic Symptom Rating Scale (PSYRATS) [22] and patients were asked to describe content and relevant linguistic features of recently experienced AVH in detail. All participants were screened with medical history, physical examination, structural magnetic resonance imaging, and laboratory testing. Exclusion criteria were history of head trauma, neurological disorders, DSM-IV criteria for alcohol dependence, or positive urine test for illicit drugs on the day of admission to the hospital. A negative pregnancy test was obtained in all women before PET acquisition. All subjects provided written informed consent to participate in the study as approved by the ethics committee (CEIC) of the Hospital Clinic of Barcelona. Healthy volunteers were recruited through word of mouth; patients were invited to participate upon admission to the hospital.

### Auditory stimulation task

We collected self-reports on content and linguistic features of AVH (including language, type, number, prosody, distortion, echo, intelligibility, age and gender of the hallucinatory voices) for each patient. We asked patients to describe as many examples of AVHs as they could remember and transcribed the whole set of example AVHs (mostly short sentences and single words) for each patient, as they were described. Nine unique 30-minute recordings were created, each mimicking individual AVH features for one patient, with the participation of several male and female speakers who read statements (from single words to complex discourse) following each patient's descriptions. Specifically, each recording included all example AVHs, each separated by a silent inter-stimulus interval of 3 s, arranged in a pseudorandom order. Each example was repeated at least twice so as to create recordings of the intended length. AVH content, and therefore the content of the recordings, was largely aversive and involved in all cases critical/derogatory comments of high linguistic complexity in the 3^rd^ person. All recordings were edited so their volume had equal intensity (∼60 dB) and was clearly audible. The auditory stimulation condition consisted of binaural reproduction of the 30-minute recordings through headphones. Patients were stimulated using the recording based on their own report; control participants were randomly assigned to listen to one of the recordings.

### PET imaging protocol and data reconstruction

We acquired FDG-PET scans at rest (scan 1) and during auditory stimulation (scan 2) for both study groups. The two scanning sessions took place within a week apart (5±2 days). All subjects fasted for at least four hours before intravenous injection of ^18^F-FDG (4.7 MBq/kg of body weight). At the moment of the injection, they all had a blood glucose concentration below 130 mg/dl. From at least 10 minutes before FDG administration to 30 minutes after, all subjects were silent and rested in a dark room to avoid visual stimulation. For the auditory stimulation condition, the reproduction started five minutes before FDG injection. Image acquisition began 35 minutes after FDG injection, using a PET scanner (Advance Nxi, GE Healthcare). To minimize motion during image acquisition, the subject's head was strapped to a cushioned head rest. A 1-minute emission scan was performed to verify the correct brain position in the field of view (14.5 cm). A 20-minute emission scan and a 7-minute transmission data scan were then acquired using a two-dimensional mode and 283×336 matrix. Slice thickness was 5 mm. Image reconstruction was performed on a SUN workstation (SUN Microsystems Mountain View, California, USA), using a 128×128×35 matrix, with a voxel size of 4.29×4.29×4.25 mm^3^. The ordered-subsets expectation maximization algorithm with 28 subsets and two iterations was used for reconstruction. Attenuation correction was performed using the acquired transmission data. The standardized uptake value was calculated voxelwise for each session from tissue counts on static images, injected dose, and body weight [23].

### Data processing and statistical analysis

Preprocessing and statistical analysis of the PET images were performed in SPM5 (Statistical Parametric Mapping 5, Wellcome Department of Neurology, London, UK; http://www.fil.ion.ucl.ac.uk/spm). Images were visually inspected for motion artifacts by an expert in quality assurance of PET images. Preprocessing included manual reorientation, affine co-registration of scan 2 to scan 1, joint spatial normalization into MNI (Montreal Neurological Institute) space of both scans and smoothing with a Gaussian kernel (full-width-at-half-maximum: 12 mm).

#### Hypothesis one testing

The primary aim was to establish differences in relative glucose metabolic rate (rGMR) between hallucination-prone schizophrenia patients and healthy controls by means of a voxelwise, full-factorial analysis. We generated a 2 (Group: patients, controls) ×2 (Condition: stimulation, baseline) linear model in SPM (*hypothesis one model*). We accounted for global nuisance and gain effects by including the global covariate (in an AnCova) as one regressor per group (AnCova by Group factor) and using proportional scaling by group [24]. We assumed unequal variance of measurements and chose a relative proportional threshold masking of 0.8. The planned contrasts for this analysis were [Patient_Stimulation_ - Control_Stimulation_] and [Patient_Stimulation-Baseline_-Control_Stimulation-Baseline_].

#### Hypothesis two testing

To assess group differences in amygdalar correlations with other brain regions, we generated a second factorial model in SPM (*hypothesis two model*) with the same design described for the *hypothesis one model* except for the addition of one regressor per group corresponding to rGMR extracted from a left amygdala region-of-interest (ROI). We followed a previously described method for assessing “metabolic connectivity” using resting FDG-PET, where mean counts extracted from an ROI are used as a covariate in a regression model to find voxels where activity correlates with the ROI activity across subjects [25], [26]. This and similar methods provide information about functional organization of the brain by detecting “functional covariance networks” [27], [28], which are partially converging with functional networks detected via within-subject correlations across time points within a time-series of BOLD-fMRI signal [29]. Our focus here was on within-group patterns of amygdalar correlations and between-group differences in amygdalar correlation with auditory regions. MarsBaR toolbox version 0.42 was used to extract subject-scaled rGMR values for the left amygdala (center coordinates (mm): −25 −3 −11; radius: 10 mm) and the left auditory cortex (cluster centered at coordinates (mm): −64 −16 0) ROIs. The left amygdala ROI was defined based on group differences during stimulation (thresholded at p = 0.005, uncorrected) that fell within an anatomically predefined mask of bilateral amygdalae (see Results). This mask was created using WFU PickAtlas Tool Version 2.4 [30]. The auditory cortex ROI was functionally defined by using the left temporal cortex cluster resulting from the conjunction analysis of both groups' [Stimulation-Baseline] contrasts in the *hypothesis one model* (thresholded at p = 0.005, uncorrected). A bilateral Heschl gyri ROI was generated with WFU PickAtlas to evaluate direct effects concerning the primary auditory cortex. An exploratory aim was to assess connections between amygdala and prefrontal cortex, given importance of prefrontal-limbic connections in the pathophysiology of schizophrenia [31].

#### Control analysis of medication effects

To assess whether results from *hypothesis two* were a consequence of treatment with antipsychotic medication, we performed a control analysis within patients using their pre-treatment, acute-psychotic data [19] in addition to their post-treatment, remission data (at rest and during auditory stimulation). We used a one-way AnCova design with a state factor comprising three levels (acute AVH pre-treatment, post-treatment rest, post-treatment auditory stimulation) and one covariate corresponding to rGMR in the left amygdala ROI. The critical test here was the interaction between state and amygdalar correlations. The absence of a significant interaction would represent a failure to detect state-dependent changes in amygdalar connectivity and would thus support the notion that amygdalar connectivity does not change with treatment (or cognitive state).

Statistical parametric t maps resulting from contrasts in the linear regression models were thresholded at a height of p_uncorrected_<0.005 and extent of 10 adjacent voxels. Of the surviving findings, only clusters significant at p_FWE_<0.05 (family-wise error-corrected) according to Gaussian Random Field Theory or voxels significant at p_FDR_<0.05 (false discovery rate-corrected) are reported. Reported effects for findings related to the primary auditory cortex in which we applied small-volume correction survived a more stringent cutoff of p_FWE_<0.05 at the voxel level. Local-maxima coordinates were transformed into Talairach space using nonlinear registration [32] and anatomically labeled based on the nearest gray-matter point using the Talairach Daemon atlas (http://www.talairach.org/). All coordinates are presented in MNI space and images are presented in neurological convention (left is left).

## Results

There were no significant differences in sociodemographical features between the study groups (mean age ± s.d., 25.33±5.5 in patients, 28.14±4.6 in controls; t_15_ = 0.46, p = 0.69; % women, 44% in patients, 50% in controls, p = 1, Fisher's exact test). All participants were Caucasian. Patients were maintained on the same dose of risperidone (range: 4−9 mg/day) throughout the current experiment. After a minimum of one-year follow-up in our outpatient clinic, schizophrenia diagnosis was confirmed in all patients. Before symptom remission, patients reported marked (“clear evidence of voices that occur frequently”) to severe (“clear evidence of voices that occur almost daily”) AVH. All patients reported commenting hallucinatory voices with critical/derogatory content. Table 1 summarizes the clinical characteristics of patients during their acute psychotic episode, prior to treatment and remission. None reported hallucinations during FDG uptake on scans 1 or 2 of the current study.

**Table 1:** Clinical characteristics of AVH patients during the acute psychotic episode (n = 9).

	Mean ± SD
Duration of untreated psychosis (months)	24±23
PANSS Positive Scale (P) Total	26.33±4.15
PANSS P1. Delusions	5.44±1.01
PANSS P2. Conceptual disorganization	4.33±1.41
PANSS P3. Hallucinatory behavior	4.88±0.92
PANSS P4. Excitement	3.33±1.32
PANSS P5. Grandiosity	2.11±1.16
PANSS P6. Suspiciousness/persecution	4.66±0.86
PANSS P7. Hostility	2.22±1.56
PANSS Negative Scale (N) Total	20.66±5.44
PANSS General Psychopathology Scale (G) Total	45.00±8.20
PSYRATS	
1. Frequency	2.88±0.78
2. Duration	2.11±0.78
3. Location	2.33±1.22
4. Loudness	2.00±0.86
5. Beliefs about origin of voices	2.55±1.13
6. Amount of negative content	2.88±0.92
7. Degree of negative content	3.22±0.97
8. Amount of distress	3.22±0.44
9. Intensity of distress	3.22±0.66
10. Disruption to life	3.11±0.33
11. Controllability	3.44±0.72

PANSS: Positive and Negative Syndrome Scale. PSYRATS: Psychotic Symptom Rating Scale.

### Hypothesis one results

**Figure 1:**
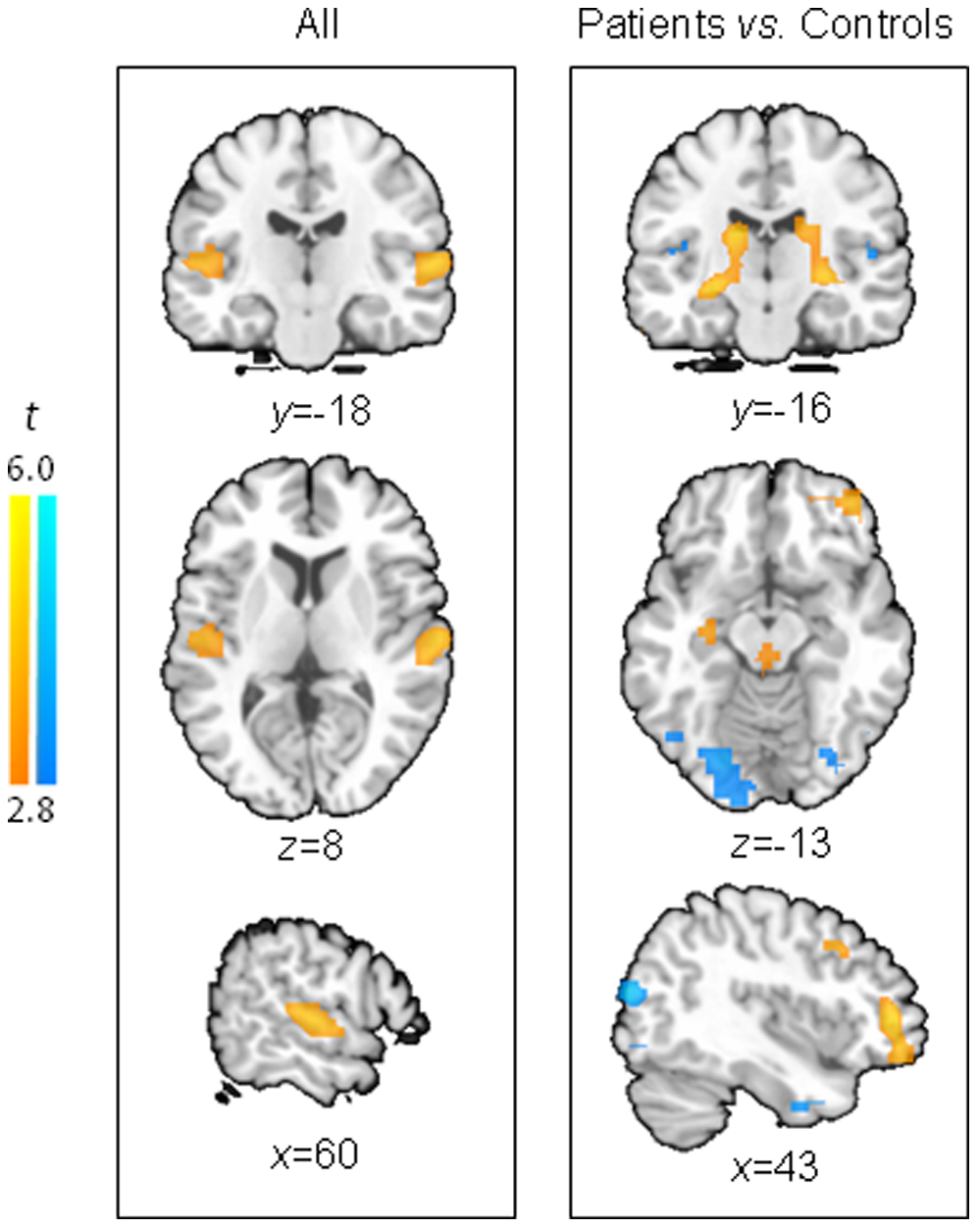
Group effects of auditory stimulation on brain metabolism (rGMR). *Left panel*: *t* maps show increased brain metabolism in the superior temporal cortex during the auditory stimulation condition relative to the resting condition in all participants (including patients and controls). *Right panel*: during auditory stimulation, patients showed increased metabolism in mesolimbic regions and decreased metabolism in fusiform gyrus compared to controls. Maps are thresholded at p = 0.005 and 10 adjacent voxels. Orange colors represent a relative increase in metabolic activity; blue colors represent a relative decrease in metabolic activity.

As hypothesized, patients showed greater activation in limbic and paralimbic regions, as well as in temporal cortex and other brain regions, relative to healthy controls in the stimulation condition (**Figure 1** **,** **Table 2** **, and** **Figure S1**): patients had hyperactivity bilaterally extending from the hippocampus and thalamus through to the amygdalae, along with hyperactivity in the left orbitofrontal cortex, right superior temporal cortex and brainstem-cerebellar vermis (overlapping with the periaqueductal gray and other brainstem nuclei, all t_28_>3.66, p_FDR_<0.050 at the voxel level), and hypoactivity in the fusiform gyri (t_28_>5.04, cluster extent>158 voxels, p_FWE_<0.050 at the cluster level) relative to controls. In addition, we observed a substantial overlap in the regions of the temporal cortex that responded to stimulation across the study groups (right superior temporal gyrus, conjunction analysis of Stimulation-Baseline in both groups at p = 0.005, uncorrected; and bilateral superior temporal gyri, average effect of Stimulation-Baseline across all participants, **Figure 1**). The main effect of condition in both groups was detected at trend level in the left temporal cortex (t_28_ = 5.10, cluster extent = 143 voxels, p_FWE_ = 0.063 at the cluster level, local maxima xyz coordinates (mm): −64 −4 −4). The Patient_Stimulation-Baseline_-Control_Stimulation-Baseline_ contrast did not yield significant findings, nor did the contrast between the groups at rest ([Patient_Baseline_-Control_Baseline_]).

**Table 2:** Group differences in rGMR during auditory stimulation.

Cluster Location	Cluster-level statistics			Voxel-level statistics					Peak Coordinates (MNI)	Peak Location
	pFWE	k	p, unc.	pFWE	pFDR	*t*	Z	p, unc.	x, y, z (mm)	
**Patients > controls**										
Right middle frontal gyrus	0.029	177	0.002	0.081	0.038	5.36	4.41	<0.001	36 40 4	Middle frontal gyrus (BA 10)
				0.431	0.042	4.48	3.86	<0.001	40 44 −16	Middle frontal gyrus (BA 11)
				0.928	0.063	3.71	3.32	<0.001	20 48 −12	Anterior cingulate (BA 10)
Left and right cerebellum and brainstem	0.073	137	0.006	0.093	0.038	5.29	4.37	<0.001	0 −36 −24	Left anterior lobe
				0.948	0.069	3.65	3.27	0.001	−4 −56 −28	Left anterior lobe, nodule
				1	0.137	2.97	2.74	0.003	8 −68 −28	Right anterior lobe, uvula
Left thalamus, parahippocampal and hippocampal gyri and amygdala	0.034	169	0.003	0.094	0.038	5.28	4.36	<0.001	−16 −20 16	Thalamus, lateral posterior nucleus
				0.127	0.038	5.14	4.28	<0.001	−28 −20 −12	Parahippocampal and hippocampal gyri
Right thalamus, globus pallidus	0.006	248	<0.001	0.128	0.038	5.14	4.28	<0.001	24 −16 −4	Globus pallidus
				0.394	0.042	4.54	3.89	<0.001	16 −32 12	Thalamus, pulvinar nucleus
Right middle frontal gyrus	0.778	37	0.117	0.244	0.038	4.81	4.07	<0.001	16 32 44	Middle frontal gyrus (BA 8)
				0.993	0.092	3.37	3.06	0.001	16 40 28	Middle frontal gyrus (BA 9)
Left middle frontal gyrus	0.981	15	0.308	0.266	0.038	4.76	4.04	<0.001	−52 20 44	Middle frontal gyrus (BA 8)
	0.412	67	0.041	0.603	0.044	4.24	3.69	<0.001	36 16 36	–
Right superior temporal gyrus	0.993	11	0.384	0.63	0.045	4.2	3.67	<0.001	76 −24 4	Superior temporal gyrus
**Controls > patients**										
Right occipital lobe	0.043	159	0.003	0.136	0.095	5.11	4.26	<0.001	40 −88 16	Middle occipital gyrus (BA 19)
				0.506	0.102	4.37	3.78	<0.001	44 −84 0	Inferior occipital gyrus (BA 18)
				0.848	0.13	3.88	3.44	<0.001	24 −76 −12	Right cerebellum, posterior lobe, declive
Left occipital lobe and fusiform gyrus	0.008	238	0.001	0.154	0.095	5.05	4.22	<0.001	−36 −80 −8	Fusiform gyrus (BA 19)
				0.867	0.132	3.85	3.42	<0.001	−36 −96 8	Occipital gyrus (BA 18)
				0.894	0.133	3.79	3.38	<0.001	−16 −100 −20	–

Table shows local maxima more than 8 mm apart. k: number of adjacent voxels per cluster. unc.: uncorrected. BA: Brodmann area.

### Hypothesis two results

**Figure 2:**
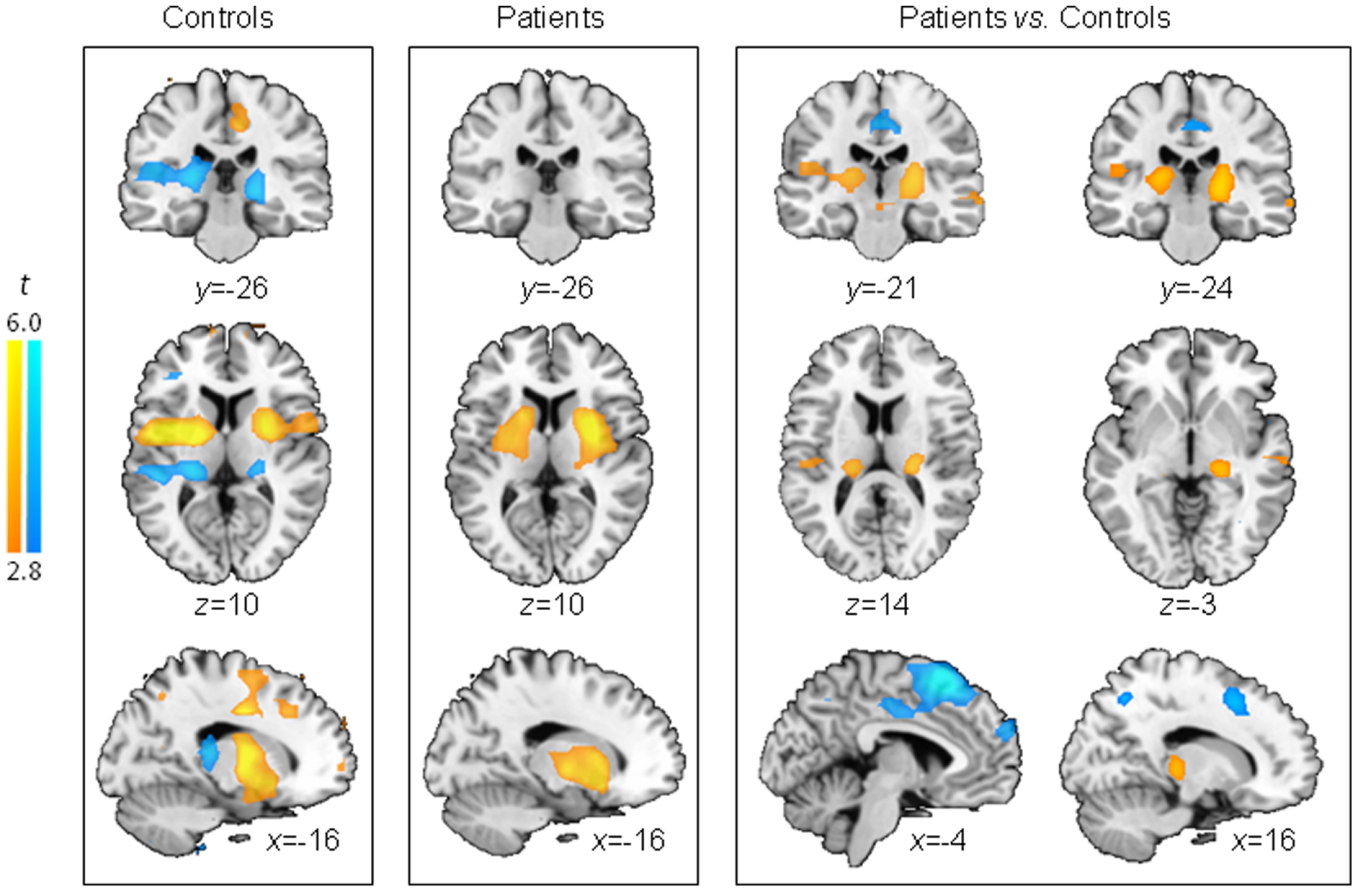
Functional correlations of left amygdala across participants. Brain regions in which rGMR correlates positively (orange colors) or negatively (blue colors) with the amygdala rGMR across participants: controls (*left*), patients (*middle*), and difference between the groups (*right*). Compared to controls, patients had stronger correlations (orange) between the amygdala and the thalamus-hippocampus and weaker correlations (blue) with the medial prefrontal cortex across conditions. Maps are thresholded at p = 0.005 and 10 adjacent voxels.

Both groups shared local correlations of the left amygdala bilaterally with the hippocampus, striatum (mostly putamen), globus pallidus, and orbitofrontal cortex, as well as connections with the contralateral amygdala (conjunction analysis of amygdala correlations for both groups; t>6.90, cluster extent: 392 (left) and 273 (right), cluster-level p_FWE_≤0.002). However, differences between the two groups were also prominent. Group-by-amygdala interaction effects were most prominent in the medial wall of the brain, encompassing bilateral supplementary motor, medial prefrontal, and middle cingulate cortices and precuneus, but were also present in bilateral hippocampi, posterior thalami, right inferior frontal, and right middle posterior temporal cortices (F_1,26_>20.02, voxel-level p_FDR_<0.050; **Figure 2** **,** **Table 3** **and** **Figure S2**). These interactions were explained by weaker correlations in patients relative to controls of the left amygdala with medial prefrontal, precuneus and right middle temporal cortex (all t_26_>3.90, p_FDR_<0.050 at the voxel level) and stronger correlations in patients relative to controls of the left amygdala with posterior regions of the thalamus (overlapping with the medial geniculate nucleus, part of the auditory thalamus, **Figure S3**) and hippocampus (t_26_ = 5.25, cluster extent = 144, p_FWE_ = 0.047 at cluster-level, local maxima xyz coordinates (mm): −20 −28 4). Moreover, stronger amygdala-auditory cortex interaction was detected in patients with schizophrenia relative to controls (small-volume-corrected to the Heschl gyri ROI; t_26_ = 3.97, voxel-level p_FWE_ = 0.038, p_FDR_ = 0.051, and t_26_ = 3.41, voxel-level p_FWE_ = 0.112, p_FDR_ = 0.056, for left and right Heschl's gyri, respectively).

**Table 3:** Group differences in amygdalar correlations.

Cluster Location	Cluster-level statistics			Voxel-level statistics					Peak Coordinates (MNI)	Peak Location
	pFWE	k	p, unc.	pFWE	pFDR	*t*	Z	p, unc.	x, y, z (mm)	
**Patients > controls**										
Left thalamus, superior temporal cortex	0.597	49	0.066	0.717	0.752	4.23	3.62	<0.001	−24 −28 4	Left thalamus
Right thalamus, hippocampus, middle temporal cortex	0.219	88	0.018	0.84	0.752	4.03	3.49	<0.001	20 −32 8	Right thalamus
				0.978	0.752	3.62	3.2	0.001	32 −36 −16	
**Controls > patients**										
Left frontal lobe	<0.001	709	<0.001	0.023	0.012	6.26	4.77	<0.001	−12 20 60	Superior frontal gyrus (BA 6)
				0.141	0.017	5.32	4.28	<0.001	−8 4 52	Cingulate gyrus (BA 24)
				0.772	0.036	4.15	3.57	<0.001	−36 8 56	Middle frontal gyrus (BA 6)
Left precuneus, right cuneus	0.08	126	0.006	0.123	0.017	5.4	4.32	<0.001	0 −68 32	Precuneus (BA 31)
				0.339	0.019	4.81	3.99	<0.001	8 −64 40	Cuneus (BA 7)
Left posterior cingulate, occipital lobe	0.61	48	0.068	0.468	0.024	4.6	3.85	<0.001	−44 −60 20	Occipital lobe (BA 19)
				0.999	0.082	3.29	2.96	0.002	−28 −60 16	Posterior cingulate (BA 30)
Left claustrum	0.736	39	0.097	0.81	0.038	4.09	3.53	<0.001	−32 −4 24	Claustrum
Left insula	0.818	33	0.124	0.824	0.04	4.06	3.51	<0.001	−48 −8 4	Insula (BA 13)
Right insula	0.954	20	0.224	0.829	0.04	4.05	3.5	<0.001	48 12 24	Insula (BA 13)
Right insula	0.722	40	0.093	0.853	0.042	4.01	3.47	<0.001	48 4 −8	Insula (BA 13)
Left frontal lobe	0.279	79	0.024	0.867	0.044	3.98	3.45	<0.001	−16 60 32	Superior frontal gyrus (BA 9)

Table shows local maxima more than 8 mm apart. k: number of adjacent voxels per cluster. unc.: uncorrected. BA: Brodmann area.

### Control analysis of medication effects

**Figure 3:**
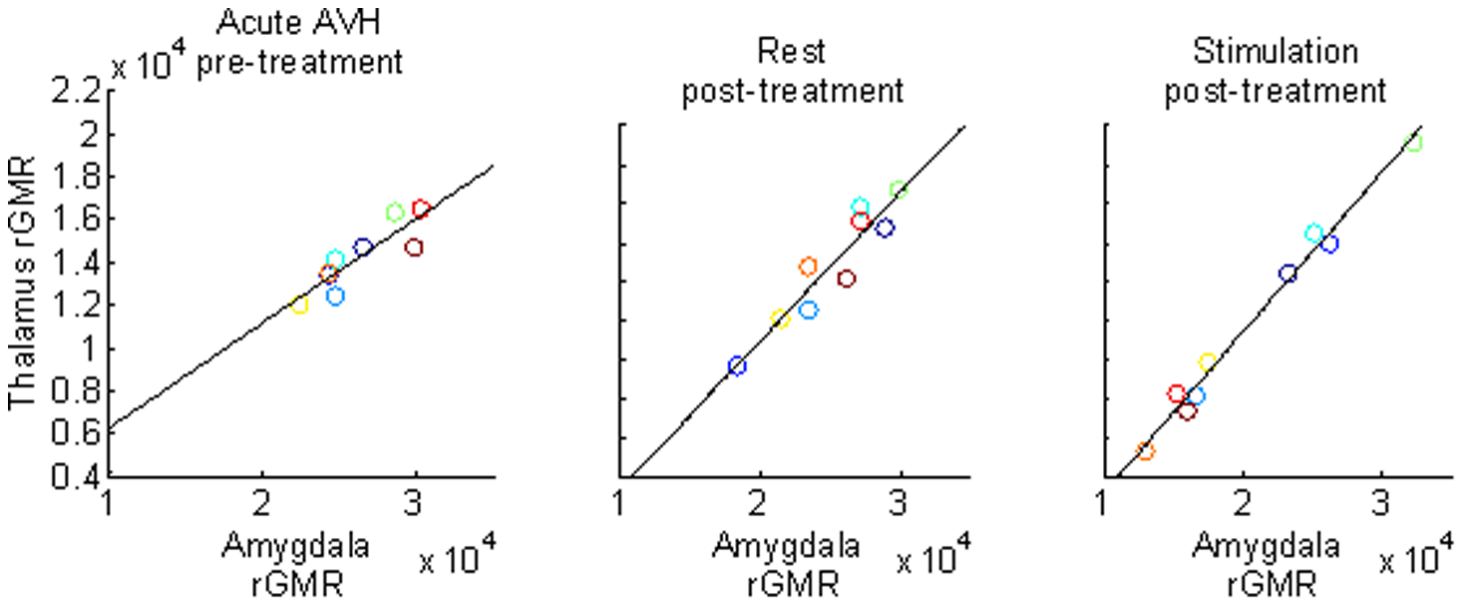
Functional correlations of rGMR between left amygdala and posterior (auditory) thalamus in patients. Scatterplots show positive correlations between the rGMR in the amygdala ROI and the thalamus cluster with significant group effects (Figure 2) across all three states: before treatment, in the acute phase of AVH (*left panel*), post-treatment at rest (*middle panel*), and post-treatment during auditory stimulation (*right panel*). Each patient is represented by the same color in the three panels.

Finally, we performed a post-hoc analysis of state-dependent effects using pre-treatment data from the same AVH patients during acute psychosis [19] to assess whether our results concerning *hypothesis two* were driven by medication (see Methods). We tested interactions between state and amygdalar correlations in a whole-brain analysis within the patient group. No regions showed significant state-by-amygdala effects, even using a lenient threshold of p = 0.05, uncorrected (**Figure 3** shows scatter plots for the main cluster of group differences in Figure 2, corresponding to the posterior thalamic regions that overlap with auditory thalamic nuclei). This result suggests that our findings of abnormal amygdalar connections in patients were not likely driven by medication effects or task conditions.

## Discussion

Our findings demonstrate mesolimbic and auditory hyperactivity in remitted hallucination-prone patients with schizophrenia during exposure to hallucination-like verbal stimuli. Amygdalar connectivity in these patients was weaker than normal for medial prefrontal regions and abnormally strong for limbic and sensory auditory regions. These findings support previous molecular [8], anatomical [33], and neuroimaging [11], [34], [35] evidence of mesolimbic involvement in the pathophysiology of positive symptoms, and fit well within a framework of previously hypothesized mechanisms for the generation of such symptoms [2], [5]. Dysfunctional limbic to sensory cortex interactions thus arise as a potential mechanism involved in the core perceptual symptoms of schizophrenia.

Previous imaging studies have used auditory stimulation in the form of emotional words in chronic hallucinators and hallucination-like stimulation in remitted patients. These studies have shown that patients with schizophrenia have increased hemodynamic responses in limbic regions (including amygdala, cingulate gyrus, and orbitofrontal cortex), among other brain regions, during auditory stimulation [14], [15]. Sanjuan et al. [14] specifically reported increased limbic activation (in amygdala, cingulate gyrus, and orbitofrontal cortex) along with increased activation in the superior temporal cortex in chronic hallucinators under verbal stimulation. Our results parallel and extend these findings to sustained limbic and auditory responses, supporting the contention that limbic and auditory dysfunctions may be involved in the pathophysiology of AVH and may account for the emotional features of AVH. To our knowledge, the current study is the first to show abnormalities in accumulated neural activity of mesolimbic regions under auditory stimulation mimicking AVH, as measured with FDG-PET.

Our design was most similar to Ait Bentaleb et al.'s [15] in that our stimulation condition consisted of reproducing speech stimuli similar in content to the hallucinations that patients experienced. A potential pitfall of this approach, however, is that patients could be more familiar with stimulus content than controls. Our finding of limbic hyperactivity in patients could thus represent an increased familiarity with the stimuli in this group rather than abnormalities related to the processing of aversive stimuli *per se*. However, several arguments weigh against this interpretation. The stimuli in our task were similar (but not verbatim) to the AVH content as reported by the patients and participants had not listened to these particular recordings prior to the experiment. Additionally, most patients had frequent AVH that changed rapidly in content and other characteristics (rather than stereotypical, repetitive AVH), so their reports, and therefore the stimuli presented during stimulation, might not have represented particularly memorable or salient instances of AVH but instead AVH that happened to occur close in time to the clinical interview. Finally, San Juan et al. [14] reported similar findings of stronger hemodynamic responses to novel auditory stimuli with emotional, aversive content in limbic regions including the amygdala in chronic hallucinators versus healthy controls. Critically, the stimuli used in this study were not directly based on reports from the study patients and therefore familiarity could not explain group differences in their fMRI measures. These arguments thus provide some support for the argument that limbic hyperactivity represents an abnormal processing of aversive stimuli in patients with AVH rather than an effect of familiarity. Nonetheless, we do acknowledge the possibility of a differential effect of familiarity with the recordings content in our group findings as part of the unbridgeable experiential differences between patients and controls. Although we believe that our current focus on individual characteristics of AVHs represents a particular strength of this study, future research on phenomenology should strive to circumvent such experiential differences across groups while keeping a focus on individual experiences.

Our analyses revealed abnormal functional connections of the amygdala in patients with AVH. Specifically, the left amygdala displayed abnormally strong connections to the auditory thalamus and the auditory cortex in patients compared to controls. The left amygdala is central to enhancing the perception of aversive verbal material in healthy individuals [13], likely through its interactions with the auditory system [36]. Thus, increased interactions between the amygdala and the auditory system might render aversive stimuli particularly salient or make the perception of neutral stimuli acquire emotional characteristics or salience normally associated with aversive stimuli. Another possible account relates to our finding that patients had abnormally weak connections between amygdala and areas of the prefrontal cortex involved in self-referential processing [37], [38] and top-down control of emotions [39]. In line with studies showing a prefrontal modulation of emotions via prefrontal-limbic interations [39], [40], increased amygdalo-auditory connectivity in patients could reflect a failure in top-down regulation of amygdala function by the prefrontal cortex. This latter interpretation is also consistent with the extensive literature emphasizing prefrontal abnormalities [41], [42] and abnormal prefrontal-amygdala interactions [31] in schizophrenia.

In addition to these notable differences between patients with schizophrenia and healthy controls, we also found meaningful similarities between the groups. First, both patients and controls engaged a similar region within the temporal cortex in response to auditory stimulation. This common response may imply that the functional organization of the auditory cortex is preserved at least to some extent in patients. Second, in addition to the excess of short-range intra-limbic connections found in patients, the two groups exhibited overlapping connectivity between the amygdala and limbic-paralimbic regions. This relatively preserved functionality of the auditory cortex in conjunction with the abnormal connectivity between the amygdala and auditory regions, may suggest that the key disturbance underlying AVH lies in amygdalo-auditory circuits that modulate activity in the temporal cortex rather than in the auditory cortex itself, consistent with current views of hallucination pathophysiology [43]. Dysfunctions in a modulatory circuit of the auditory cortex involving limbic regions and the thalamus have been recently linked to tinnitus [44], [45], [46], a positive symptom accompanied by some of the emotional features of AVH. Our results suggest that auditory positive symptoms in a broad sense, including tinnitus and AVH, might share dysfunctions in a common modulatory circuit involving limbic regions that are normally responsible for noise attenuation in the auditory system. Because we showed that the abnormal interactions between the amygdala and auditory regions were present before and after treatment and during rest and stimulation, these interactions do not seem to be induced by treatment. Although there is no direct histopathological evidence confirming such altered connectivity in patients with AVH, growing molecular evidence points to aberrant excitatory outputs from the amygdala in persons with schizophrenia [8].

There are some relevant limitations to the current study. The small sample size could make our analyses susceptible to a type II error, thus underestimating true differences between groups. While FDG-PET imaging was suitable to achieve our goal of investigating sustained neural activation, its low temporal resolution prevented an assessment of the effects of linguistic and other time-varying features of the verbal stimuli. Additionally, our study did not include a comparison group of schizophrenic patients without AVH, and this prevents us from drawing conclusions specific to hallucination-prone patients. With regard to *hypothesis two*, our analyses assume fixed group effects of amygdala connectivity and therefore do not account for individual variability. Future studies should seek to infer causality regarding the relationship between dysfunctional limbic connectivity and AVH, and aim to overcome these methodological drawbacks to better understand the amygdalar influence on auditory-cortex function in schizophrenia.

In sum, our study on AVH replicates previous reports of abnormally elevated neural activity in limbic regions to aversive speech stimuli. In addition, we uncovered abnormal interactions between the amygdala and auditory regions of the cortex and thalamus that persisted after symptom remission. We suggest that an abnormal modulation of the auditory cortex by a limbic-thalamic circuit might underlie the generation of emotional AVH. Further research on limbic-auditory interactions and their role in AVH is warranted.

## Supporting Information

Figure S1 **Axial view of group effects of auditory stimulation on brain metabolism (rGMR).** The *t*-statistic map is thresholded at p = 0.005 and 10 adjacent voxels and overlaid onto a single-subject T1 scan (MNI Colin brain). Hot colors represent increased rGMR in patients relative to controls. Cold colors represent increased rGMR in controls relative to patients.
(TIF)

Figure S2 **Axial view of group differences in functional correlations of left amygdala.** The *t*-statistic map is thresholded at p = 0.005 and 10 adjacent voxels and overlaid onto a single-subject T1 scan (MNI Colin brain). Hot colors represent regions of increased connectivity with the amygdala in patients relative to controls. Cold colors represent regions of increased connectivity with the amygdala in controls relative to patients.
(TIF)

Figure S3 **Orthogonal view of group differences in functional correlations of left amygdala.** Compared to controls, patients had stronger correlations (orange) between the amygdala and the thalamus-hippocampus (same notation as in Figure 2 of the main text). The white circles correspond to the medial geniculate nucleus (MGN) of the auditory thalamus. Note the overlap of the significant cluster of increased amygdalar connectivity for patients (orange) with the MGN. Maps are thresholded at p = 0.005 and 10 adjacent voxels.
(TIF)
