# Supplementary figures and images for: Brain Metabolism during Hallucination-Like Auditory Stimulation in Schizophrenia
Source: PLoS One. 2014 Jan 8;9(1):e84987. doi: 10.1371/journal.pone.0084987 (PMC3885666; doi:10.1371/journal.pone.0084987)

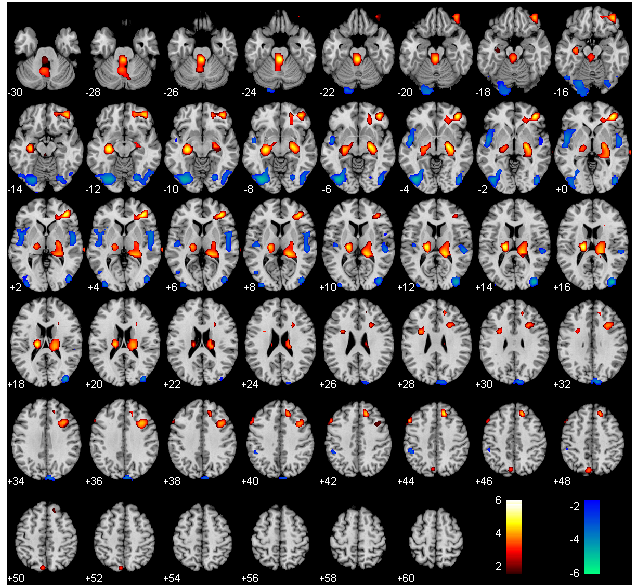

Supplement: Figure S1 — Axial view of group effects of auditory stimulation on brain metabolism (rGMR). The t-statistic map is thresholded at p = 0.005 and 10 adjacent voxels and overlaid onto a single-subject T1 scan (MNI Colin brain). Hot colors represent increased rGMR in patients relative to controls. Cold colors represent increased rGMR in controls relative to patients. (TIF) [file pone.0084987.s001.tif]

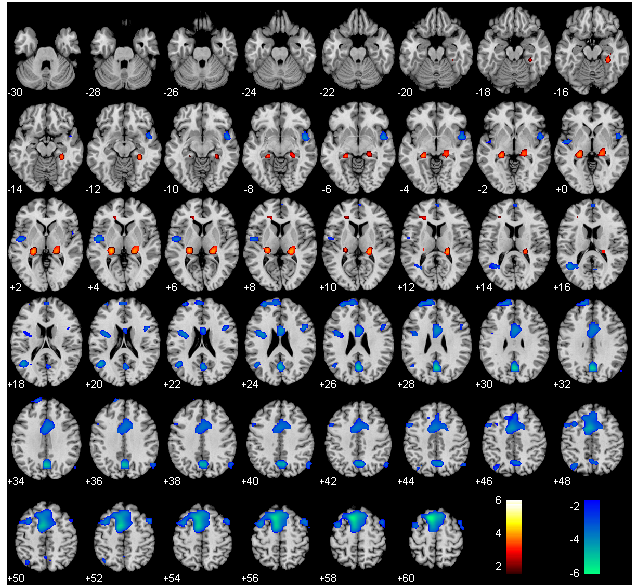

Supplement: Figure S2 — Axial view of group differences in functional correlations of left amygdala. The t-statistic map is thresholded at p = 0.005 and 10 adjacent voxels and overlaid onto a single-subject T1 scan (MNI Colin brain). Hot colors represent regions of increased connectivity with the amygdala in patients relative to controls. Cold colors represent regions of increased connectivity with the amygdala in controls relative to patients. (TIF) [file pone.0084987.s002.tif]

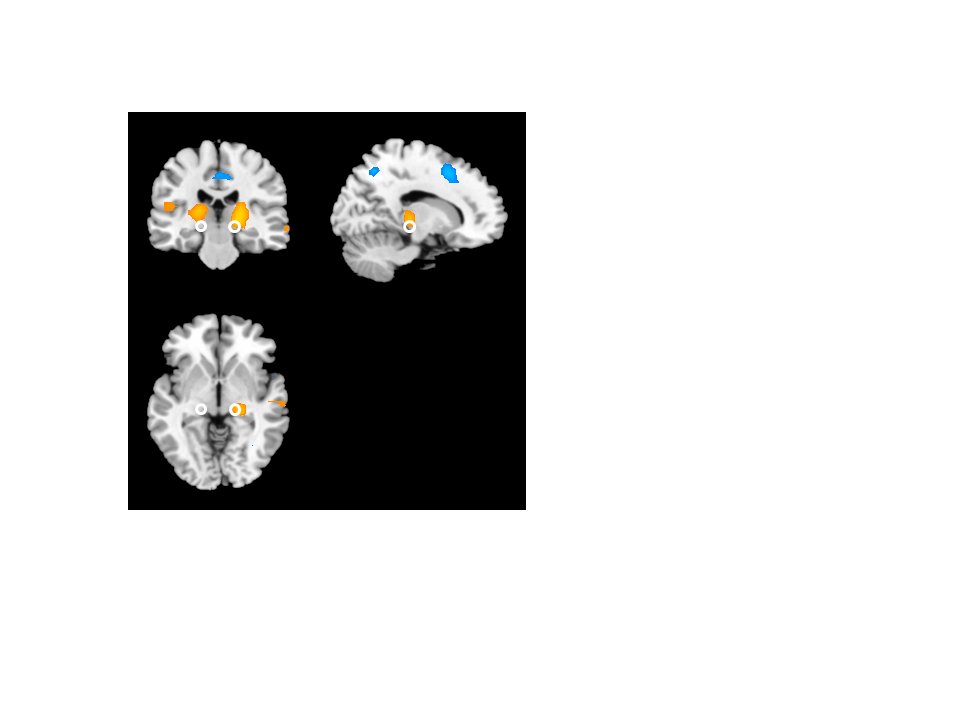

Supplement: Figure S3 — Orthogonal view of group differences in functional correlations of left amygdala. Compared to controls, patients had stronger correlations (orange) between the amygdala and the thalamus-hippocampus (same notation as in Figure 2 of the main text). The white circles correspond to the medial geniculate nucleus (MGN) of the auditory thalamus. Note the overlap of the significant cluster of increased amygdalar connectivity for patients (orange) with the MGN. Maps are thresholded at p = 0.005 and 10 adjacent voxels. (TIF) [file pone.0084987.s003.tif]
